# The Effect of Duck Targeted Lighting Programs on Productivity, Health, and Welfare

**DOI:** 10.3390/ani16101440

**Published:** 2026-05-08

**Authors:** Parker B. Watson, Emily Jiral, Melinda Grimes, Gregory S. Archer

**Affiliations:** Department of Poultry Science, Texas A&M University, College Station, TX 77845, USA; pbwatson9997@tamu.edu (P.B.W.);

**Keywords:** duck, dynamic lighting, LED, photoperiod, light spectrum, welfare, corticosterone, tonic immobility, fear, stress

## Abstract

This study evaluated the impact of a dynamic LED lighting program specifically tailored to Pekin ducks (DDYNs) compared to a conventional commercial lighting program (CON) on growth performance, health, stress, and fear responses. A total of 960 Pekin ducks were reared across two replicates. Production parameters including body weight, average daily gain, feed intake, and feed conversion ratio were recorded. Health was assessed via tibia breaking strength, bone ash content, complete blood cell count, and villus height to crypt depth ratio. Stress was evaluated through physical asymmetry, heterophil-to-lymphocyte ratio, and plasma corticosterone concentration, while fear was measured using tonic immobility and inversion tests. No significant differences were observed in production or health parameters between treatments (*p* > 0.05). However, ducks reared under the DDYN program exhibited significantly lower stress indicators, including reduced corticosterone (*p* = 0.011), lower heterophil-to-lymphocyte ratios (*p* = 0.045), and decreased asymmetry (*p* = 0.015). Fear responses were also significantly reduced under DDYN lighting (*p* < 0.001). These findings suggest that species-tailored dynamic lighting programs can meaningfully improve Pekin duck welfare without compromising production performance.

## Simple Summary

Pekin ducks are an important part of global poultry production, yet little research has explored how lighting conditions during rearing affect their well-being. Ducks see light differently than humans and other poultry, so lighting programs designed for chickens may not be ideal for them. This study compared a standard commercial lighting program to a dynamic lighting program specifically designed for Pekin ducks, which gradually adjusted brightness and color over the 35-day growing period to better match the species’ natural visual needs. A total of 960 ducks were raised across two trials, and researchers measured growth, bone health, blood indicators of stress, and behavioral tests of fear. Ducks raised under both lighting programs grew equally well and had similar body weights and feed efficiency. However, ducks raised under the duck-specific lighting program showed significantly lower levels of stress, as measured by stress hormones in the blood and physical markers of chronic stress. These ducks also displayed less fearful behavior when tested. These results suggest that tailoring lighting programs to the specific visual biology of Pekin ducks can meaningfully reduce stress and fear without sacrificing growth performance, offering producers a practical way to improve animal welfare.

## 1. Introduction

Just as broilers are sensitive to aspects such as photoperiod, wavelength, and light intensity, so are Pekin ducks [1]. Through the utilization of retinal receptors and “extraretinal receptors” in combination with oil droplets, which prevent an overabundance of spectral overlap, ducks have the ability to perceive lighting in a way humans cannot [2,3]. Although the effects of lighting have been thoroughly studied in broilers, ducks have received limited attention. Duck meat makes up a large part of global poultry production [3], which should make it a priority for research. Duck production also differs from other poultry production [4]. A firm understanding of how different lighting aspects impact the rearing process of Pekin ducks could lead to new innovations in the field.

Pekin ducks are descendants of the wild Mallard ducks, a breed of fowl that is predominantly found on or in proximity to a body of water, and their primary food sources consist of vegetation and small vertebrates found under the surface of the water [1]. Ducks that forage underwater have been shown to have relatively fewer R-type droplets in their cone photoreceptors in comparison to chickens and other species of poultry, possibly due to longer wavelengths being absorbed quickly on the water’s surface [5]. It is hypothesized that ducks may use a dense population of short wavelength-sensitive cones similarly to how chickens and other species of poultry use long wavelength-sensitive cones to perceive their environment [6]. Further investigation is needed to understand how aspects of lighting like photoperiod, intensity, and spectrum impact the behavior and physiology of ducks.

Studies have shown that lighting is an abiotic stressor [6] and can influence the growth [7] and development [8] of ducks just like chickens. One study by Campbell [1] found that contrary to broilers, ducks raised under a blue light had reduced body weight (BW) at every age until final weighing compared to ducks reared under red light or white lighting [1]. However, a more recent study by Hefnawy et al. [9] found that no differences in production parameters were observed between ducks reared under red, blue, yellow, and white lighting. Photoperiod has also been shown to have an impact on production parameters, with a study by House et al. concluding that ducks reared under a 20L:4D photoperiod had more efficient feed conversion ratios compared to ducks reared under a 16L:8D photoperiod [10]. Another important aspect of proper bird development is bone health [11]. Like other poultry grown quickly for meat purposes, ducks can be more susceptible to underperformance and injury without proper skeletal development [8]. A study by Tonissen et al. [12] found that ducks raised under a pulsed alternating wavelength LED program had increased early interstitial growth in femurs and tibias, increased bone mineral content in femurs, and wider and increased geometrical bone mechanical properties in the humeral bone compared to ducks reared under a traditional light program, possibly implying a relationship between lighting and duck bone development.

Fear and stress have been shown to play a role in the production and welfare of ducks, with increased levels in either category potentially hindering physiological and behavioral processes. Fear response can be measured using several assessments, including tonic immobility and inversion. This combination of tonic immobility [TI] and inversion tests has been used in multiple studies to determine fear susceptibility in poultry by assessing natural behavioral responses [13,14]. Studies have found Pekin duck stress and fear to be influenced by different aspects of lighting, including photoperiod [10], intensity [2], and wavelength [10,15,16,17]. However, with very little research done into the effects of lighting on duck fear and stress, further investigation is required to learn how producers, as well as animals, can benefit from these effects.

Although some research has been reported regarding lighting and its impact on Pekin ducks, there has been almost no research into the effects of a transitioning lighting program, especially one specifically tailored to Pekin ducks during the rearing process [18]. The objective of this experiment was to determine the effects of a lighting program specifically tailored to Pekin ducks on stress, fear, health, and growth. It is hypothesized that rearing ducks under a dynamic lighting program specifically tailored to the species will increase production and improve development while minimizing levels of stress and fear, compared to ducks reared under a traditional lighting program.

## 2. Materials and Methods

### 2.1. Birds, Housing and Experimental Design

Straight-run Pekin ducks were reared in two separate environmentally controlled rooms, each devoid of natural light to eliminate the influence of external photoperiods. Each room contained 12 floor pens, measuring 1.22 × 1.83 m. Twenty ducks were reared in each pen, totaling 480 ducks per trial. The trial was replicated to ensure reliability of the data, with lighting treatments switched between rooms in the second replicate to control potential room-related variability, resulting in a total sample size of 960. Ducks were reared with ad libitum access to feed and water, and temperature and humidity were maintained based on the Maple Leaf Farms INDUX Meat Duck Handbook. One treatment was equipped with a standard commercial LED lighting set to a conventional lighting program (CON). The other treatment utilized a dynamic LED lighting program specifically tailored to Pekin ducks (DDYN), designed to mimic lighting more naturalistic to the species.

**Figure 1:**
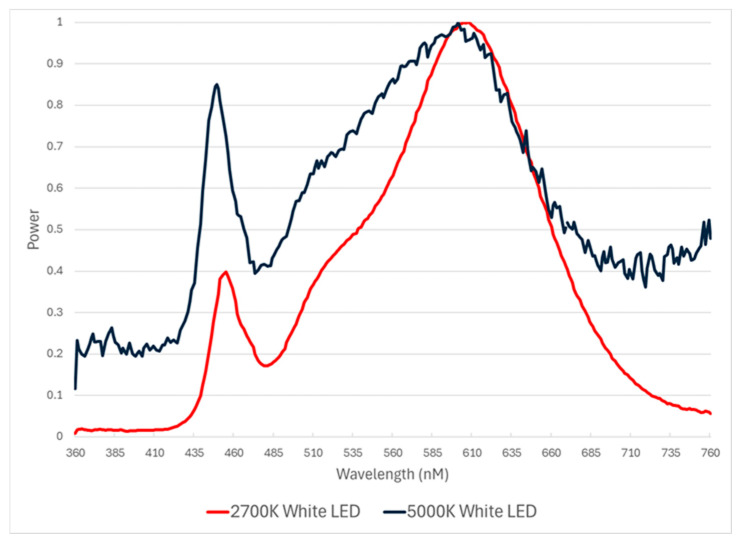
Spectral output of CON lighting treatment: 2700K d 0–5 and 5000K d 6–end of trial.

The CON treatment room contained 6 white LED bulbs (ONCE by Signify, Plymouth, MN, USA) fixtures per room, located directly over pens 3 m above the floor and controlled by a single dimmer and timer. A summary of the CON lighting treatment is provided in Table 1 and Figure 1.

**Figure 2:**
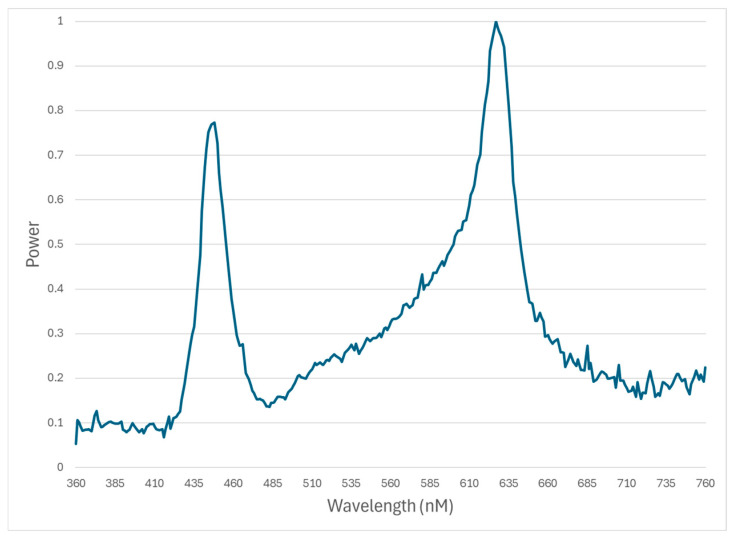
Spectral output of DYNY lighting treatment during whole trial.

The DDYN treatment room contained 6 dynamic LED light bulbs (Once by Signify, USA) installed directly above pens 3 m above the floor and controlled using the Interact Agriculture (Once by Signify, Plymouth, MN, USA.) application. A schedule of photoperiod and lux for the DDYN treatment is presented in Table 2. The spectral output of the DDYN treatment is presented in Figure 2.

Birds received a commercial duck starter diet at the beginning of the trial (day 0), with 12 kg of feed allocated per pen. On day 15, the remaining feed in each pen was weighed and recorded to determine feed intake. At that point, birds were transitioned to a grower diet, which they received until day 35, the end of the trial. Remaining feed was again collected and weighed at the conclusion of the study to assess total consumption. Feed was offered ad libitum throughout the trial, with additional feed provided as needed to ensure continuous access. All feed was produced by the Texas A&M feed mill. Diet composition can be found in Table 3.

### 2.2. Growth and Feed Conversion

Birds were weighed at the start and the conclusion of the trial on day 35 to determine average bird weight gain. Feed was weighed at distribution throughout the trial and was weighed back at transitions in the feed phase to calculate feed intake and feed conversion ratio. Feed intake was calculated by subtracting leftover feed at the end of the starter and grower phases from total feed allocated. The feed conversion ratio was determined by dividing feed given by the pen weights. Mortality was collected daily and weighed so that feed conversion could be corrected for mortality losses.

### 2.3. Tibia Breaking Strength and Ash Content

On day 35, both the left and right tibias were extracted from one bird in a pen at random. The sex of the bird was not recorded. After ethnicization via cervical dislocation, the tibias were removed and placed in labeled sample bags for further processing the following day. Tibia ash was determined by removing the fibula, muscle and connective tissue, and the bones were dried at 100 °C for 12 h before defatting in diethyl ether for 8 h and air drying. De-fatted tibias were dried again at 100 °C for 12 h and then ashed in ceramic crucibles at 600 °C for 24 h. Breaking strength was measured by the 3-point bending test using the TA XT Plus100 Texture Analyzer (Texture Technologies Corporation, Scarsdale, NY, USA).

### 2.4. Blood Measurements: Heterophil/Lymphocyte Ratio, Complete Blood Cell Count, and Plasma Corticosterone Concentration

On day 35 of each trial, one bird per pen was selected at random for heterophil-to-lymphocyte ratio (HL), corticosterone analysis, and complete blood cell count (CBC). The sex of the bird was not recorded. Blood (4–5 mL) was collected from the branchial wing vein of each broiler. Immediately after collection, a small drop of blood was placed on a glass slide and smeared for the heterophil-to-lymphocyte (HL) ratio. All remaining blood collected was then split between tests, injecting half into a plasma separation gel and lithium heparin vacutainer and placing it in an ice bath for temporary storage. The other half was injected into a lavender-top tube containing ethylenediaminetetraacetic acid to prevent coagulation of the blood and sent off for a complete blood cell count. Following collection of all blood samples, all vacutainers for corticosterone concentrations were spun down in a centrifuge at 4000 RPM for 15 min to separate plasma and blood cells. Once separated, blood plasma samples were poured into a 2 mL microcentrifuge tube and stored at –19C until further analysis could be performed. A hematology staining kit was used for staining blood smear slides used for the heterophil-to-lymphocyte ratio (HL).

Plasma corticosterone concentration from each sample was assessed using commercially available ELISA kits. Inter- and intra-assay %CV were both under 5%. To determine heterophil-to-lymphocyte ratios of each sample, a single layer of blood cells on a glass slide was separated, stained, and placed under a microscope (Omax DCE-2, Kent, WA, USA) for observation. Moreover, 40X magnification using an oil immersion lens was utilized for observation of slides. A keystroke counter (SEOH B4001-5LC, Navasota, TX, USA) was used to record the number of heterophils and lymphocytes observed in an area of the blood smear without overlapping cells. Heterophils and lymphocytes were counted until a total of 100 cells were recorded. Elevated plasma corticosterone and heterophil-to-lymphocyte ratios can be indications of higher stress susceptibility in poultry [19,20].

### 2.5. Physical Asymmetry

On day 35 of each trial, 5 birds per pen were selected at random for measurement of three bilateral traits on the feet. The sex of the bird was not recorded. Using a calibrated Craftsman IP54 Digital Caliper (Sears Holdings, Hoffman Estates, IL, USA), the middle toe length, metatarsal length, and metatarsal width were measured for both the right and left legs. The composite asymmetry score was calculated by taking the sum of the absolute value of the left minus right of each trait, then divided by the total number of traits. Thus, the formula for this trial would be (|L-R|MTL + |L-R|ML + |L-R|MW)/3 = composite asymmetry score.

### 2.6. Tonic Immobility

Tonic immobility was collected on the final day of the trial. Five birds per pen were selected at random to collect. The sex of the bird was not recorded. Tonic immobility measurements. Birds were handled with extreme care in order to not interfere with measurements. The subjects were flipped over with their abdomen facing upward and placed within half a large U-shaped cradle lined with cloth. Once placed on their back, the researcher’s hand remained on their breast for approximately 25 s to induce tonic immobility in the animal. The subject had to remain immobile for at least 10 s to register as a viable attempt. If the bird were to flip over within the first 10 s period, this would be marked as an attempt. The subject was then reset for another attempt. If three attempts were made inconclusive, a time of 0 s was recorded. Assuming the subject passed this first 10 s period of immobility, the first head movement was then monitored and recorded. Finally, the subject was monitored for latency to right themselves, for which the final measurement of time was collected. The greater time needed for correction is an indication of greater fear in the broilers being tested.

### 2.7. Inversion

Similarly to tonic immobility, inversion is another measurement of fear collected from birds at the conclusion of the study. Once again, five birds per pen were selected. Birds were randomly selected and caught. The sex of the bird was not recorded. Once caught, birds were held by their legs and then inverted until they ceased to flap or reach a maximum time of 30 s. Each instance of inversion on a bird was recorded via video camera to be analyzed later for measurements of intensity. Trained observers then observed the footage and recorded the number of flaps, as well as the amount of time the flapping occurred. From these measurements, we were able to determine wing flap intensity by dividing the number of flaps by the duration of time in seconds that the flapping occurred. A greater fear response is represented by a greater intensity of wing flapping.

### 2.8. Statistical Analysis

All data was analyzed using Minitab software (version 22.4) via one-way ANOVA using the General Linear Model (GLM) procedure. A pen was considered the experimental unit; when multiple birds were measured within a pen, the average of the pen was used for analysis. Differences in *p* < 0.05 were considered statistically significant.

## 3. Results

### 3.1. Production and Health

Production was assessed using d35 body weight (BW), average daily feed intake (ADFI), average daily gain (ADG), and feed conversion ratio (FCR) and is presented in Table 4. No significant differences were found in BW, ADG, or FCR (*p* > 0.05).

Assessments of health included tibia bone breaking strength (BS), bone ash content (BA), complete blood cell count (CBC), and villi height to crypt depth ratio (V:C) and are presented in Table 4. No differences in BS or WBC were observed between treatments. Ducks reared under the DDYN program showed a trend for lower BA (47.4 ± 0.85%, *p* = 0.06) than those reared under the conventional program (49.29 ± 0.50%). Ducks reared under the DDYN program also showed a trend for a lower villi height to crypt depth ratio (3.75 ± 0.131, *p* = 0.058) than those reared under the CON treatment (4.16 ± 0.169).

### 3.2. Stress and Fear Response

Measurements of stress included asymmetry (ASYM), heterophil-to-lymphocyte ratio (HL), and plasma corticosterone concentration (CORT) and are presented in Table 5. Ducks reared under the dynamic lighting program had lower stress susceptibility as indicated by asymmetry, HL, and CORT (1.92 ± 0.10 mm, *p* = 0.015; 0.51 ± 0.03, *p* = 0.045; 16,628 ± 2349 pg/mL, *p* = 0.011, respectively) compared to those reared under the traditional lighting program (2.53 ± 0.23 mm; 0.61 ± 0.04; 24,870 ± 2042 pg/mL, respectively).

Fear response was measured using tonic immobility (TI) and inversion (INV) and is presented in Table 5. Ducks under the dynamic lighting program flapped (3.86 ± 0.25 flaps, *p* < 0.001) less but for a longer duration (3.36 ± 0.18 s, *p* < 0.001) during the inversion test, resulting in lower flapping intensity (1.11 ± 0.05 flaps/s, *p* < 0.001), indicating lower levels of fear compared to the ducks under the conventional lighting program (7.12 ± 0.58 flaps; 2.49 ± 0.15 s; 2.86 ± 0.14 flaps/s, respectively). Dynamic lighting ducks also had a shorter latency to first head movement (66.3 ± 10.33 s, *p* = 0.001) and time to right (166.9 ± 17.16 s, *p* < 0.001) during the tonic immobility test compared to those reared under the traditional lighting program (123.6 ± 14.45 s; 277.6 ± 21.46 s, respectively), indicating lower levels of fear.

## 4. Discussion

The objective of this study was to evaluate the effects of a dynamic LED lighting program specifically tailored to Pekin ducks (DDYN) on production performance, health, stress, and fear compared to a conventional commercial lighting program (CON). While Pekin ducks constitute a significant portion of global poultry meat production [3], research into species-specific lighting strategies for ducks remains limited relative to broiler chickens. The present findings indicate that while the DDYN program did not significantly alter production or health parameters, it substantially reduced indicators of stress and fear, supporting the hypothesis that a species-tailored lighting program can improve Pekin duck welfare.

No significant differences in body weight, average daily gain, average daily feed intake, or feed conversion ratio were observed between ducks reared under the DDYN and CON (*p* > 0.05). These results are consistent with the findings of Hefnawy et al. [7], who reported no differences in production measures between ducks reared under red, blue, yellow, and white lighting. However, they contrast with earlier work by Campbell et al. [1], who found reduced body weight in ducks reared under blue light compared to red or white light. The discrepancy may be attributed to differences in experimental design; whereas Campbell et al. [1] utilized static monochromatic lighting, the DDYN program in the present study employed a gradually transitioning spectrum and photoperiod designed to approximate more naturalistic conditions. House et al. [10] demonstrated that photoperiod influences feed conversion in ducks, with a 20L:4D schedule yielding more efficient ratios than 16L:8D. In the current study, both treatments converged on similar photoperiods by the end of the trial (16L:8D for DDYN and 18L:6D for CON by day 28), which may partially explain the lack of production differences. Taken together, these results suggest that while specific wavelengths or extreme photoperiods may influence duck growth, the dynamic transitioning approach used here maintained production equivalence with conventional lighting.

Health parameters, including tibia breaking strength, bone ash content, white blood cell count, and villus height to crypt depth ratio, did not differ significantly between treatments, though several trends approached significance. Ducks under the DDYN program exhibited numerically higher tibia breaking strength (33,279 vs. 26,693 g, *p* = 0.31) but lower bone ash content (47.4 vs. 49.29%, *p* = 0.06). The trend toward reduced bone ash in DDYNs warrants further investigation, as bone mineralization is a critical component of skeletal health in rapidly growing meat ducks [8,11]. Tonissen et al. [12] reported that ducks reared under a pulsed alternating wavelength LED program demonstrated increased early interstitial bone growth and enhanced geometrical bone properties, suggesting that lighting spectrum may influence bone development through mechanisms beyond simple mineral deposition. The near-significant reduction in villus height to crypt depth ratio observed in DDYNs (3.75 vs. 4.16, *p* = 0.058) may reflect differences in intestinal epithelial turnover; however, both values remain within the range considered normal for healthy poultry, and the functional significance of this difference remains unclear. Future studies with larger sample sizes may be needed to determine whether these trends represent biologically meaningful effects of dynamic lighting on duck health.

The most compelling findings of this study were the significant reductions in stress indicators among ducks reared under the DDYN program. Plasma corticosterone concentrations were significantly lower in DDYNs compared to CON ducks (16,628 vs. 24,870 pg/mL, *p* = 0.011), representing a 33% reduction. This finding aligns with the established understanding that corticosterone serves as a primary glucocorticoid stress indicator in poultry [19,20] and suggests that the dynamic lighting environment was perceived as less stressful by the birds. Similarly, the heterophil-to-lymphocyte ratio was significantly lower in DDYNs (0.51 vs. 0.61, *p* = 0.045), providing a complementary hematological marker of reduced chronic stress. Previous work by House et al. [10,16] demonstrated that wavelength and photoperiod can independently influence stress responses in Pekin ducks. The present study extends these findings by demonstrating that a program integrating gradual transitions in both spectrum and photoperiod can achieve meaningful stress reduction. The significantly lower composite asymmetry scores in DDYNs (1.92 vs. 2.53 mm, *p* = 0.015) further support reduced developmental stress, as fluctuating asymmetry is widely regarded as an indicator of developmental instability resulting from environmental stressors during growth [13].

Fear responses, as measured by tonic immobility and inversion tests, were consistently and significantly reduced in ducks reared under the DDYN program. During the tonic immobility test, DDYNs exhibited shorter latency to first head movement (66.3 vs. 123.6 s, *p* = 0.001) and latency to right (166.9 vs. 277.6 s, *p* < 0.001), indicating reduced fear susceptibility. These results are consistent with the interpretation that longer durations of tonic immobility reflect heightened fear states in poultry [13,14]. Similarly, the inversion test revealed that DDYNs displayed significantly fewer wing flaps (3.86 vs. 7.12 flaps, *p* < 0.001) and markedly lower flapping intensity (1.11 vs. 2.86 flaps/s, *p* < 0.001), both indicative of a diminished fear response. Interestingly, while DDYNs flapped less frequently, they flapped for a longer duration (3.36 vs. 2.49 s, *p* < 0.001), which may suggest a calmer, more sustained but less panicked behavioral response compared to the short, intense flapping bursts observed in CON ducks. House et al. [2,10,15,16,17] previously demonstrated that individual lighting parameters such as photoperiod, intensity, and wavelength each influence fear and stress in Pekin ducks. The present study suggests that combining these elements into a cohesive, transitioning program amplifies the welfare benefits beyond what any single lighting parameter may achieve alone.

The mechanisms underlying the observed stress and fear reductions likely involve the unique visual physiology of Pekin ducks. As descendants of Mallard ducks that forage underwater, Pekin ducks possess relatively fewer R-type oil droplets in their cone photoreceptors compared to chickens [5], and may rely more heavily on short wavelength-sensitive cones to perceive their environment [7]. The DDYN lighting program, which featured a greater spectral peak producing a red/white color and incorporated gradual transitions in intensity and photoperiod, may have provided a more comfortable photic environment for duck-specific visual processing. Additionally, the gradual stepdown in light intensity and photoperiod during the first 10 days of the DDYN program, as opposed to the abrupt transitions in the CON, may have reduced the physiological stress associated with sudden environmental changes. This approach aligns with the broader principle that gradual environmental transitions are less disruptive to animal welfare than abrupt ones [18].

In summary, the results of this study demonstrate that a dynamic lighting program tailored to the visual biology of Pekin ducks can substantially reduce stress and fear indicators without compromising growth performance or health. These findings have practical implications for the duck production industry, as they suggest that species-specific lighting represents a feasible, non-invasive management strategy for improving welfare. Given the growing emphasis on animal welfare in poultry production and the expanding global demand for duck meat, further refinement and commercial validation of duck-targeted lighting programs should be a priority for future research.

## 5. Conclusions

This study demonstrated that a dynamic LED lighting program specifically tailored to Pekin ducks significantly reduced stress and fear without negatively impacting growth performance or health. Ducks reared under the DDYN program exhibited significantly lower plasma corticosterone concentrations, heterophil-to-lymphocyte ratios, and composite asymmetry scores, collectively indicating reduced physiological and developmental stress. Fear responses were also markedly lower, as evidenced by shorter tonic immobility durations and reduced wing flap intensity during inversion testing. Importantly, these welfare improvements were achieved with no significant differences in body weight, feed conversion, or average daily gain, suggesting that the dynamic program does not compromise productivity.

These findings support the hypothesis that lighting programs designed around the unique visual biology of Pekin ducks can meaningfully enhance welfare outcomes. Given the limited research on species-specific lighting for ducks relative to broiler chickens, this study provides foundational evidence that transitioning lighting programs warrant further investigation and potential adoption within the commercial duck industry. Future research should explore the individual contributions of spectral composition and transition scheduling, examine effects on processing and meat quality traits, and validate these results under commercial-scale conditions to facilitate practical implementation.

## Figures and Tables

**Table 1:** Duck control (CON) lighting treatment summary with photoperiod, lux, and Kelvin.

Day	Photoperiod	Lux	Kelvin
0	24L:0D	25	3000
1–5	23L:1D	25	3000
6–20	16L:8D	5	5000
21–27	17L:7D	5	5000
28–35	18L:6D	5	5000

**Table 2:** Photoperiod and lux schedule of the dynamic DDYN lighting program.

Day	Lux	Time On	Time Off	Photoperiod
1	63.1	0:30	23:00	23L:1D
2	63.1	0:30	23:00	23L:1D
3	55.8	0:54	22:31	22.13L:1.87D
4	48.5	1:18	22:03	21.25L:2.75D
5	41.3	1:43	21:35	20.38L:3.62D
6	34	2:07	21:07	19.5L:4.5D
7	26.8	2:31	20:39	18.63L:5.37D
8	19.5	2:56	20:11	17.75L:6.25D
9	12.3	3:20	19:43	16.88L:7.12D
10–35	5	3:45	19:15	16L:8D

**Table 3:** Ingredient composition of the basal diets for the starter (fed d 0–14) and grower (fed d 15–35) diet phases ^1^.

Ingredient	Starter (%; d 0–14)	Grower (%; d 15–35)
Corn	43.37	55.23
Soybean meal	39.70	27.28
Soy Oil	5.91	7.90
Wheat Middlings	6.02	6.01
DL-Methionine, 98%	0.36	0.27
L-Lysine	0.01	0.08
Limestone	2.67	1.18
Monocalcium phosphate	1.25	1.32
Salt	0.42	0.42
Trace Mineral Mix ^2^	0.05	0.05
Vitamin Premix ^3^	0.25	0.25

^1^ Pellet binder (calcium lignosulfonate) was added to the grower diet at a rate of 2.72 g/kg feed. ^2^ Trace mineral premixes added at this rate yield the following per kilogram: 13.33 g manganese, 13.33 g zinc, 13.33 g iron, 1.56 g copper, 0.09 g iodine, a minimum of 1.39 g calcium and a maximum of 1.93 g calcium. Calcium carbonate was used as a carrier. ^3^ Vitamin premixes added at this rate yield the following per kilogram: 36,741.67 IU vitamin A, 12,860 IU vitamin D3, 151.67 IU vitamin E, 435.17 mg choline, 153 mg niacin, 67.33 mg D-pantothenic acid, 23.83 mg pyridoxine, 19.83 mg riboflavin, 9.78 mg thiamin, 5.83 mg folic acid, 1.83 mg biotin, and 0.07 mg vitamin B12.

**Table 4:** Comparison of production and health parameters between DDYN and CON treatments (mean ± SE).

Treatment	CON	DDYN	*p*-Value
d 35 Body Weight3 (kg)	3.21 ± 0.03	3.20 ± 0.05	0.94
Average Daily Gain (kg/d)	0.09 ± 0.01	0.09 ± 0.01	0.94
Average Daily Feed Intake (kg/b/d)	0.13 ± 0.01	0.13 ± 0.01	0.31
Feed Conversion Ratio	1.60 ± 0.01	1.62 ± 0.02	0.28
Tibia Breaking Strength (g)	26,693 ± 1316	33,278 ± 6284	0.31
Bone Ash Content (%)	49.29 ± 0.50	47.4 ± 0.85	0.06
White Blood Cell Count (%)	18.97 ± 1.10	19.29 ± 1.37	0.86
Villi Crypt Ratio	4.16 ± 0.169	3.75 ± 0.131	0.06

**Table 5:** Comparison of results for fear and stress susceptibility for DDYN and CON lighting treatments (mean ± SE).

Treatment	CON	DDYN	*p*-Value
Corticosterone (pg/mL)	24,870 ± 2042	16,628 ± 2349	0.011
Heterophil-to-lymphocyte ratio	0.61 ± 0.04	0.51 ± 0.03	0.045
Composite asymmetry score (mm)	2.53 ± 0.23	1.92 ± 0.10	0.015
Latency to right (s) during tonic immobility	277.6 ± 21.5	166.9 ± 17.2	<0.001
Latency to first head movement (s) during tonic immobility	123.6 ± 14.5	66.3 ± 10.3	0.001
# Attempts to induce during tonic immobility	1.47 ± 0.06	1.65 ± 0.08	0.17
Number of flaps during inversion (flaps)	7.12 ± 0.58	3.86 ± 0.25	<0.001
Duration of flapping (s) during inversion	2.49 ± 0.15	3.63 ± 0.18	<0.001
Intensity flapping during inversion (flaps/s)	2.86 ± 0.14	1.11 ± 0.05	<0.001

## Data Availability

The original contributions presented in this study are included in the article. Further inquiries can be directed to the corresponding author.

## Abbreviations

The following abbreviations are used in this manuscript:

ADFI	Average Daily Feed Intake
ADG	Average Daily Gain
ASYM	Asymmetry
BA	Bone Ash
BS	Breaking Strength
BW	Body Weight
CBC	Complete Blood Cell Count
CON	Control (Conventional Lighting Program)
CORT	Corticosterone
D	Dark
DDYN	Duck Dynamic (Lighting Program)
ELISA	Enzyme-Linked Immunosorbent Assay
FCR	Feed Conversion Ratio
GLM	General Linear Model
HL	Heterophil-to-Lymphocyte Ratio
IACUC	Institutional Animal Care and Use Committee
INV	Inversion
L	Light
LED	Light-Emitting Diode
LSD	Least Significant Difference
ML	Metatarsal Length
MTL	Middle Toe Length
MW	Metatarsal Width
TI	Tonic Immobility
V:C	Villus Height to Crypt Depth Ratio
